# Highly Sensitive Flexible Pressure Sensors Enabled by Mixing of Silicone Elastomer With Ionic Liquid-Grafted Silicone Oil

**DOI:** 10.3389/frobt.2021.737500

**Published:** 2021-09-20

**Authors:** Zhaoqing Kang, Yi Nie, Liyun Yu, Suojiang Zhang, Anne Ladegaard Skov

**Affiliations:** ^1^ Danish Polymer Center, Department of Chemical and Biochemical Engineering, Technical University of Denmark, Kgs. Lyngby, Denmark; ^2^ CAS Key Laboratory of Green Process and Engineering, Beijing Key Laboratory of Ionic Liquids Clean Process, State Key Laboratory of Multiphase Complex Systems, Institute of Process Engineering, Chinese Academy of Sciences, Beijing, China

**Keywords:** dielectric elastomer, ionic liquids, high-permittivity, grafting, pressure sensor, high sensitivity

## Abstract

Developing highly sensitive flexible pressure sensors has become crucially urgent due to the increased societal demand for wearable electronic devices capable of monitoring various human motions. The sensitivity of such sensors has been shown to be significantly enhanced by increasing the relative dielectric permittivity of the dielectric layers used in device construction via compositing with immiscible ionic conductors. Unfortunately, however, the elastomers employed for this purpose possess inhomogeneous morphologies, and thus suffer from poor long-term durability and unstable electrical response. In this study, we developed a novel, flexible, and highly sensitive pressure sensor using an elastomeric dielectric layer with particularly high permittivity and homogeneity due to the addition of synthesized ionic liquid-grafted silicone oil (denoted LMS-EIL). LMS-EIL possesses both a very high relative dielectric permittivity (9.6 × 10^5^ at 10^−1^ Hz) and excellent compatibility with silicone elastomers due to the covalently connected structure of conductive ionic liquid (IL) and chloropropyl silicone oil. A silicone elastomer with a relative permittivity of 22 at 10^−1^ Hz, Young’s modulus of 0.78 MPa, and excellent homogeneity was prepared by incorporating 10 phr (parts per hundreds rubber) of LMS-EIL into an elastomer matrix. The sensitivity of the pressure sensor produced using this optimized silicone elastomer was 0.51 kPa^−1^, which is 100 times higher than that of the pristine elastomer. In addition, a high durability illustrated by 100 loading–unloading cycles and a rapid response and recovery time of approximately 60 ms were achieved. The excellent performance of this novel pressure sensor suggests significant potential for use in human interfaces, soft robotics, and electronic skin applications.

## Introduction

Flexible pressure sensors which generate well-defined output signals from a given pressure have recently been in increasing demand for flexible and wearable electronic device applications such as human interfaces (Guo et al., 2019; Nie et al., 2019; Jeon et al., 2020), electronic skins (Boutry et al., 2018; Zeng et al., 2019; Bi et al., 2020), and soft robotics (Amit et al., 2019; Xie et al., 2021). Based on their working mechanisms, pressure sensors can be classified as piezoresistive (Laukhina et al., 2010; Qiu et al., 2016; Sencadas et al., 2020), capacitive (Hwang et al., 2021), piezoelectric (Deutz et al., 2017; Gao et al., 2019), and iontronic (Zhu et al., 2018; Bai et al., 2020) devices. Because they are characterized by high sensitivity, low power consumption, fast response, and are also cheap to manufacture, capacitive pressure sensors attract the most interest out of all these sensor types (Li et al., 2020; Tripathy et al., 2021).

A capacitive pressure sensor consists of a flexible dielectric layer sandwiched between two compliant electrodes to create a flexible capacitor capable of capacitive changes in response to changes in applied pressure. A high-permittivity and soft dielectric layer is crucial to enhancing the sensor’s potential for capacitive change (sensitivity), which depends on the relative dielectric permittivity (ε_r_) of the dielectric layer, the distance between electrodes, and the electrode area. Polydimethylsiloxane (PDMS) elastomers have been widely used as dielectric layers in capacitive pressure sensors because of their excellent flexibility, elasticity, and thermal stability. However, the sensitivity (∼0.01 kPa^−1^) of pressure sensors based on common PDMS elastomers is insufficient to recognize human motions such as gripping and breathing. This low sensitivity is due to the low dielectric permittivity (ε_r_ ∼3) and high Young’s modulus (Y∼1 MPa) of silicone elastomers (Zang et al., 2015; Atalay et al., 2018; Skov and Yu, 2018). However, the ability to recognize low pressure (1–10 kPa)—i.e., the range covering common intra-body pressures, breathing and heart-beating (Li et al., 2020)—is a necessary feature of pressure sensors that are to be attached to human skin to feel a mechanical stimulus. Thus, in order to prepare suitable wearable electronics, a pressure sensor with a sensitivity of 0.1–1 kPa^−1^ and linear response to external stimuli must be developed.

One efficient method for improving the sensitivity of flexible capacitive sensors is to form a layered microstructure of its dielectric layer (Ruth et al., 2020). Due to the decrease in overall modulus consequent upon forming a microstructure that includes e.g., air voids, a large deformation can be achieved under a small pressure. However, most microstructure preparation processes, such as chemical etching or photolithography (Kim et al., 2020; Ruth et al., 2020), are expensive and complex, in addition to being impractical for large-scale industrial production.

Another approach for increasing pressure sensor sensitivity is to enhance the ε_r_ of the dielectric layer by compositing with various high-permittivity additives. Inorganic additives, such as titanium oxide and carbon nanotubes, are the most commonly used for this purpose (Yu and Skov, 2015; Wang et al., 2018; Lee et al., 2019). A significant amount of additive is generally required to improve the ε_r_ of the PDMS composites, however, which can lead to some undesired side effects due to increased loading, such as poor additive dispersion and high dielectric loss. In addition, due to the inherently rigid nature of inorganic additives, the PDMS composites may suffer energy losses due to internal friction between particles and void generation during deformation, resulting in decreased flexibility and mechanical instability during practical use. Furthermore, increased rigidity can reduce the gains in sensitivity achieved due to improved permittivity.

The inclusion of ionic conductors, such as ionic liquids (ILs), into the PDMS matrix has been increasingly pursued due to the remarkable improvement in relative dielectric permittivity that ILs are able to impart to the resulting composites. This enhancing effect is a consequence of the space charge polarization of the mobile ions in the composite under the action of an electric field (Shi et al., 2021). Furthermore, like other liquid additives (Style et al., 2015; Liu et al., 2020a), ILs also function as soft additives, thereby improving the PDMS composite’s flexibility. Liu et al.(Liu et al., 2019) identified the most suitable IL (1-butyl-3-methylimidazolium hexafluoroantimonate) for platinum-catalyzed hydrosilylation out of several commonly used options, producing an optimized PDMS composite with a relative dielectric permittivity of 7.6. Methews and coworkers (Ankit et al., 2020) reported a PDMS composite with a self-enclosed IL, indicating synergistically improved electromechanical properties—i.e., a twofold increase in dielectric constant and a 100-fold decrease in Young’s modulus. Shi et al. (2021) prepared a PDMS composite with near-spherical IL droplets 0.5–1 μm in diameter which showed high relative dielectric permittivity (ε_r_ ∼20) and good mechanical stretchability (>350% strain). However, because of the inherent incompatibility of PDMS and ionic liquids, all three PDMS composites developed macroscopic phase separation inside the matrix, resulting in decreased long-term durability and instability of electrical response.

Our previous work showed that chloropropyl silicone oil can be used as a liquid additive to increase the relative dielectric permittivity and decrease the Young’s modulus of PDMS elastomers (Madsen et al., 2015; Liu et al., 2020a) due to its superior compatibility with PDMS based on “like dissolves like” rules. However, because chloropropyl silicone oil itself possesses a low relative dielectric permittivity (ε_r_ ∼11 at 10^−1^ Hz), a significant amount must be added to the elastomer matrix in order to increase the relative dielectric permittivity of the composite.

In this work, a soft additive with high relative dielectric permittivity was synthesized by combining imidazole-based IL and chloropropyl silicone oil. Flexible silicone elastomers generated via the inclusion of IL-grafted functional silicone were then prepared and analyzed. IL possesses high relative dielectric permittivity as an ionic conductor, while adding chloropropyl silicone oil to the PDMS ensures overall compatibility, making the composite elastomer a highly suitable dielectric material for pressure sensors. Due to these overall improvements in relative dielectric permittivity, softness and compatibility, the resulting pressure sensor displayed increased sensitivity and durability.

## Experimental

### Materials

[14–16%(chloropropyl)methylsiloxane]-dimethylsiloxane, LMS-152 (M_n_ = 8,750 g·mol^−1^, ∼14 chloropropyl groups per polymer) was obtained from Gelest Inc. Silanol-terminated polydimethylsiloxane, C2T (M_n_ = 22,000 g·mol^−1^) was purchased from Wacker Chemie AG. Toluene, chloroform, ethyl acetate, and 1-ethylimidazole were acquired from Sigma-Aldrich. Trimethoxylsiloxane (crosslinker) and dibutyltin diacetate (Sn catalyst) were obtained from Sika Technology AG. Silicon dioxide amorphous hexamethyldisilazane-treated particles (SIS6962.0) were acquired from Fluorochem. All products were used as received.

### Synthesis of the IL-Grafted Silicone Additive

LMS-EIL was prepared by nucleophilic substitution of chloropropyl groups with 1-ethylimidazole, then purified by extraction. For synthesis, 1-ethylimidazole (1.50 g, 15.60 mmol), LMS-152 (10.00 g, 1.14 mmol) with 15.60 mmol chloropropyl groups, and toluene (10 ml) were mixed together in a single-neck round-bottom flask. The molar ratio of 1-ethylimidazole-to-chloropropyl groups on LMS-152 was 1, as in previous work on IL synthesis (Jiang et al., 2008; Zhang et al., 2017). The reaction mixture was deoxygenated by bubbling nitrogen through it for 10 min before sealing with a rubber stopper. The reaction was then carried out at 85°C for 24 h, after which it was stopped by cooling the flask to room temperature. Purification was carried out as follows: the initial product was obtained by removing toluene in a rotary evaporator; the product was then washed three times with ethyl acetate to extract the unreacted 1-ethylimidazole; the final product was then dried in a vacuum oven for 24 h.

### Preparation of Elastomers With LMS-EIL

We reported the optimal formulation for preparing condensation-cured elastomers in a previous study on the influence of stoichiometric imbalance (Jurásková et al., 2020). C2T (5.00 g, 0.22 mmol), cross-linker (0.10 g, 0.76 mmol), and treated silica particles (10 phr) were mixed together using a dual asymmetric centrifuge at 3,500 rpm for 3 min (FlackTek Inc. DAC 150.1 FVZ-K SpeedMixer). The catalyst dibutyltin diacetate (0.5 wt%) and LMS-EIL were then added, and the mixture was speed mixed again. The uniform mixture was cast with a coating gap of 300 μm onto a polyethylene terephthalate (PET) substrate using a film applicator (3,540 bird, Elcometer, Germany). The coated samples were subsequently cured for 48 h in a humidity oven at 25°C and 80% relative humidity.

### Preparation of the Flexible Pressure Sensors

The sensing layer for the pressure sensor was comprised of pure PDMS elastomer or a PDMS composite with either LMS-152 or LMS-EIL added (dimensions: 10 mm length × 5 mm width × 1 mm thickness). Silver electrodes were then sputtered onto both top and bottom layers at a thickness of 30 nm to form a sandwiched plate capacitor using a sputter coater (Cressington, model 208HR) under vacuum conditions. Copper wires were used to connect the impedance analyzer to the specimens.

### Characterization

#### Fourier Transform Infrared Spectroscopy (FT-IR)

FT-IR spectra of the samples were acquired in the wavenumber range of 400–4,000 cm^−1^ using a Nicolet iS50 FT-IR fitted with a diamond crystal attenuated total reflection accessory (ATR). All spectra were acquired via 32 scans at a resolution of 4 cm^−1^ and were baseline corrected.

#### Proton Nuclear Magnetic Resonance (^1^H-NMR)

^1^H-NMR experiments were performed on a Bruker 300 MHz spectrometer using 100 mg ml^−1^ solutions in deuterated chloroform (CDCl_3_).

#### Gel Fraction

Gel fractions were determined via swelling experiments according to our previously reported procedure (Madsen et al., 2018). The PDMS elastomer sample (0.5 g) was immersed in solvent (CDCl_3_) for 72 h at room temperature, with the solvent being replaced every 24 h. After swelling for 3 days, the sample was removed from the solvent bath and washed several times with fresh solvent. Finally, the sample was dried for 48 h under ambient pressure at room temperature. LMS-EIL is soluble in CDCl_3_, as evidenced by the FT-IR spectra of the samples with LMS-EIL, which show that the LMS-EIL was completely removed (Supplementary Figure S1) after swelling. The gel fraction was calculated as: W_gel_ = m_e_/(m_0_-m_LME-EIL_)×100%, where m_e_ is the weight after extraction and drying, m_0_ is the initial weight of the PDMS elastomer, and m_LME-EIL_ is the weight of LMS-EIL in the initial sample. Three measurements were taken for each sample and then averaged.

#### Field Emission Scanning Electron Microscopes (FE-SEM)

FE-SEM (SU8020, Hitachi) was used to visualize the morphology of PDMS elastomers, and energy-dispersive X-ray spectroscopy (EDS) was applied to detect the element distribution profile on the surface of the samples. All samples were coated under vacuum with a 2 nm thick layer of platinum before testing.

#### Young’s Modulus, Tensile Strength and Strain at Break

Elastomers’ tensile stress-strain behavior was characterized using a material tester (Instron 3,340 materials testing system, INSTRON, United States) at room temperature. The samples were cut to 60 mm in length and 6 mm in width before being placed between two clamps and initially separated by a distance of 30 mm. The test specimen was elongated uniaxially at 10 mm min^−1^. Young’s modulus was obtained from the tangent of the stress-strain curves at 10% strain (Liu et al., 2019; Ankit et al., 2020; Shi et al., 2021). Three measurements were taken for each sample and then averaged.

#### Dielectric Relaxation Spectroscopy (DRS)

DRS testing was performed using a Novocontrol Alpha-A high-performance frequency analyzer (Novocontrol Technologies GmbH & Co, Germany) with an electrical field of about a 1 V mm^−1^, in the frequency range of 10^−1^–10^6^ Hz, at room temperature (Yu and Skov, 2015; Liu et al., 2019; Ankit et al., 2020; Liu et al., 2020a). Copper sheets were used as electrodes of functional oil and obtained elastomers to perform the DRS testing. The diameter and thickness of the tested samples were 20 and 1 mm, respectively.

#### Relative Capacitive Change

The performance of the pressure sensors was measured using a KEYSIGHT E4990A impedance analyzer (Keysight Technologies, United States) operated at a constant frequency of 200 Hz (i.e., the lowest frequency this analyzer can produce that results in stable capacitance values for the prepared elastomers) with an interfaced ElectroForce mechanical test machine (ElectroForce 3200, TA Instruments, United States) which can apply specific pressure onto the sensors.

## Results and Discussion

**SCHEME 1:**
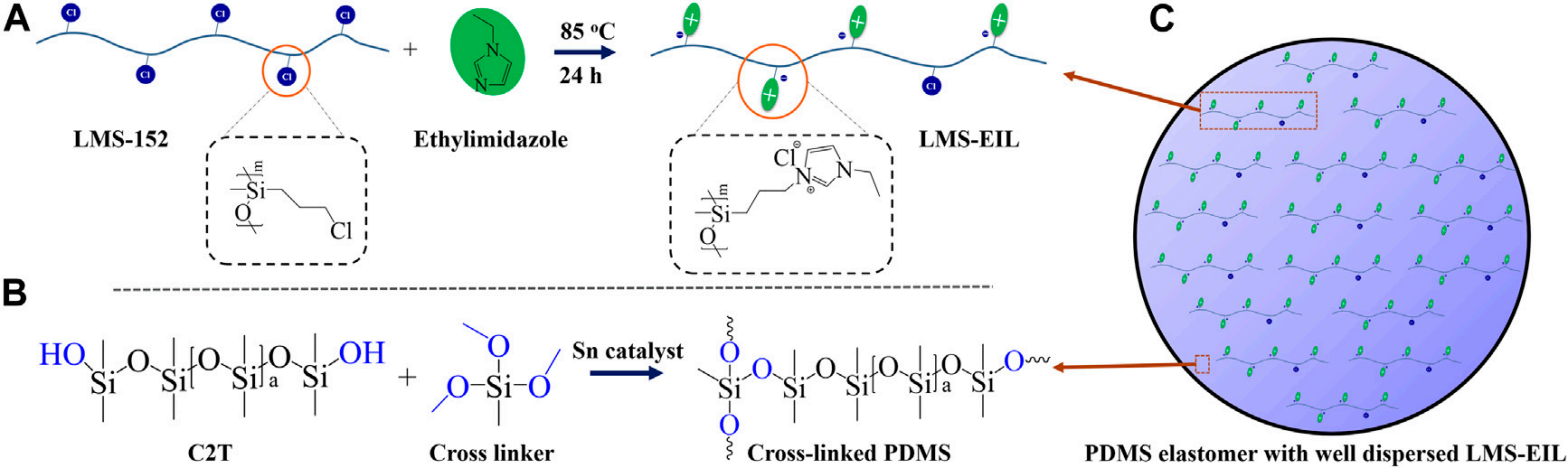
**(A)** Synthetic route to LMS-EIL through nuclear substitution of chloropropyl groups with 1-ethylimidazole; **(B)** reaction between silanol-terminated PDMS and trimethoxylsiloxane cross-linker; **(C)** cross-linked elastomer with homogeneously dispersed LMS-EIL in the matrix.

**FIGURE 1:**
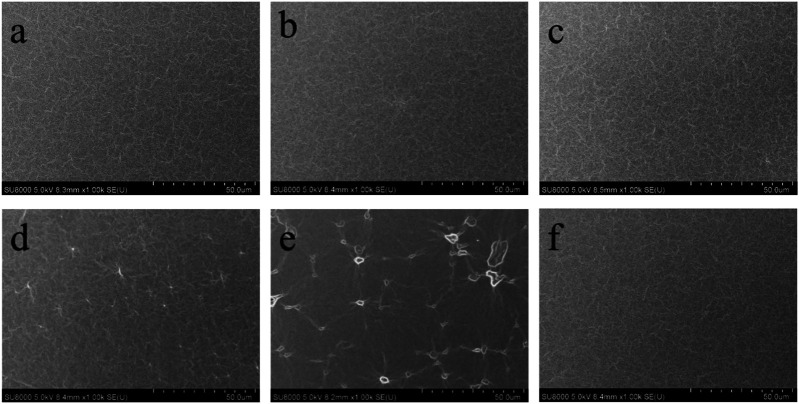
FE-SEM images of PDMS elastomers: **(A)** pure elastomer, **(B)** elastomer with 5 phr LMS-EIL, **(C)** elastomer with 10 phr LMS-EIL, **(D)** elastomer with 15 phr LMS-EIL, **(E)** elastomer with 20 phr LMS-EIL, **(F)** elastomer with 10 phr LMS-152.

IL-grafted silicone oil was synthesized by nucleophilic substitution of chloropropyl groups on LMS-152 with 1-ethylimidazole, as shown in Scheme 1A. Following the scheme illustrated in Scheme 1B, pure PDMS elastomer and PDMS elastomers with IL-grafted silicone oil were then prepared by condensation reaction between a telechelic silanol-terminated PDMS and trimethoxylsiloxane cross-linker in the presence of a tin catalyst and humidity (Jurásková et al., 2020). The resulting elastomer structure with well dispersed LMS-EIL is illustrated in Scheme 1C. The maximum content capable of maintaining a homogenous dispersion of LMS-EIL in the PDMS matrix is 10 phr, as shown in Figure 1. Although IL groups are immiscible with the PDMS matrix, the PDMS component of the LMS-EIL-modified elastomer will ensure overall compatibility, provided the LMS-EIL content is not too high.

### Properties of the IL-Grafted Silicone Oil

#### ^1^H-NMR and FT-IR Analysis of LMS-EIL

**FIGURE 2:**
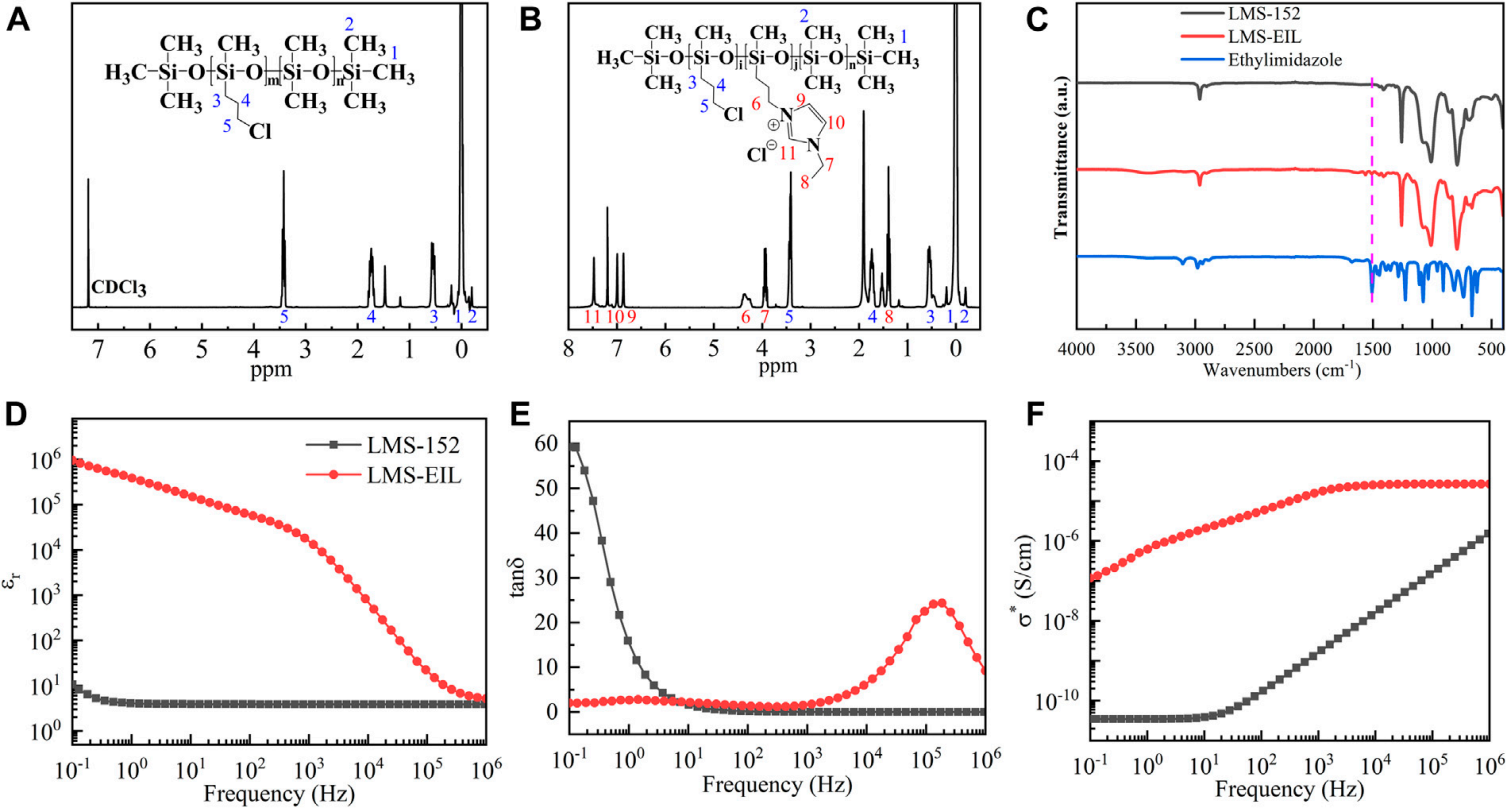
**(A)**^1^H-NMR spectrum of LMS-152; **(B)**^1^H-NMR spectrum of LMS-EIL; **(C)** FT-IR spectra of 1-ethylimidazole, LMS-EIL, and LMS-152; **(D)** relative dielectric permittivity (ε_r_) of LMS-EIL and LMS-152; **(E)** loss tangent (tanδ) of LMS-EIL and LMS-152; **(F)** complex conductivity (σ^*^) of LMS-EIL and LMS-152. The dielectric properties are measured at room temperature.

^1^H-NMR analysis was used to investigate the extent of IL grafting onto chloropropyl-silicone oil. Figures 2A,B show the spectra acquired for LMS-152 and LMS-EIL, respectively. As noted in Figure 2B the corresponding proton chemical shifts in the imidazole structure are visible at 6.86, 6.99, and 7.48 ppm (peaks 9, 10 and 11) in the spectrum. This indicates that the imidazole-based IL has been successfully grafted to the side chain of LMS-152 through nucleophilic substitution (Kim et al., 2003). The peaks at 3.41 and 4.36 ppm (peaks 5 and 6) represent the chemical shifts of protons on carbon 5 and 6, respectively, indicating the presence of original chloropropyl groups and grafted imidazole groups in the molecular structure of LMS-EIL. Since the molar ratio of 1-ethylimidazole-to-chloropropyl groups was 1 in the preparation of LMS-EIL, it is clear that partial grafting of IL onto LMS-152 also took place. The incomplete reaction may be the result of incompatibility between silicone and imidazole. The efficiency of which imidazole was incorporated into LMS-152 was evaluated from the grafting ratio (G): G = j/(i + j)×100%, where i and j represent the number of chloropropyl and imidazole groups on the molecule of LMS-EIL, respectively. Since the areas under peaks 5 and 6 are proportional to the number of chloropropyl and imidazole groups in the molecule of LMS-EIL. The grafting ratio of IL in LMS-EIL is calculated to be 30%. As shown in Figure 2C LMS-EIL displays the characteristic peaks of an imidazole ring at 1,508 cm^−1^, which can be regarded as further proof of successful IL-grafting. Nevertheless, due to the small grafting ratio, the intensities of the peaks representing imidazole groups for the molecule of LMS-EIL are relatively low.

#### Dielectric Properties

Dielectric spectroscopy of LMS-EIL was performed over the frequency range of 10^−1^–10^6^ Hz. Because LMS-EIL is used to modify PDMS elastomers, its initial relative dielectric permittivity strongly affects that of the composites, and thus also the corresponding pressure sensors’ sensitivity. Figures 2D–F show the relative dielectric permittivity (ε_r_), loss tangent (tanδ), and complex conductivity (σ^*^) of LMS-EIL and LMS-152, respectively, while the specific values of ε_r_, tanδ, and σ^*^ for both compounds at 10^−1^ and 10^6^ Hz are presented in Supplementary Table S1. The ε_r_ of LMS-EIL is approximately 9 × 10^4^ times higher compared to that of pristine chloropropyl-silicone oil at 10^−1^ Hz. This drastic increase in ε_r_ results from the presence of IL groups in the polymer structure, as IL possesses a much higher relative dielectric permittivity (Huang and Weingärtner, 2008) than silicone due to the space charge polarization of its mobile ions under electric field (Yu and Skov, 2015). As expected ε_r_ also decreases in line with increasing frequency (Figure 2D), reflecting the leveling off of polarization as dipoles fail to follow the rapid alternation of the electrical field (Weingärtner et al., 2007). While increases in relative dielectric permittivity are generally followed by large increases in loss tangent, the tanδ of LMS-EIL is nevertheless ∼30 times lower compared to that of LMS-152 at 10^−1^ Hz (Figure 2E), indicating that less dielectric absorption occurs with LMS-EIL compared to LMS-152. As shown in Figure 2F, LMS-152 exhibits typical silicone behavior, with a conductivity of 3.5 × 10^−11^ at low frequencies. However, LMS-EIL exhibits a plateau in conductivity of 2.7 × 10^−5^ at high frequencies owing ILs’ high conductivity. ILs consist of organic cations and inorganic anions, or vice versa, and therefore possess much higher conductivity than silicone (Lu et al., 2020). Our previous work on IL-modified PDMS elastomers such as 1-ethylpyridinium tetrafluoroborate (Su et al., 2020) and 1-butyl-3-methylimidazolium hexafluoroantimonate (Liu et al., 2019; Liu et al., 2020b) showed that these materials displayed slightly higher σ^*^ values than that of the pure elastomer, albeit their overall conductivity was still considered to be exceptionally low. It is therefore also possible that LMS-EIL can be used as an additive to modify PDMS elastomers despite its improved complex conductivity.

### Morphology of PDMS Elastomers

The dispersion of LMS-EIL additive within the elastomer matrix was investigated using FE-SME and FE-SEM coupled with EDS. Figure 1 shows the FE-SEM surface images of pure elastomer, elastomer with 10 phr LMS-152, and elastomers modified with different amounts of LMS-EIL. Magnified FE-SEM and optical cross sectional images of these elastomers are shown in Supplementary Figures S2, S3. The elastomers with 5 and 10 phr LMS-EIL clearly display uniform morphologies comparable to that of both the pure elastomer and the elastomer with LMS-152. Furthermore, the EDS images (Supplementary Figure S4) of the elastomer with 10 phr LMS-EIL quite clearly demonstrate the presence of N and Cl uniformly dispersed throughout the sample. However, LMS-EIL droplets are clearly visible in the elastomers with 15 and 20 phr LMS-EIL—indicating that aggregation has taken place; the size of these droplets increased with increasing LMS-EIL concentration, indicating more serious aggregation in the resultant elastomer.

### Dielectric Properties of PDMS Elastomers

**FIGURE 3:**
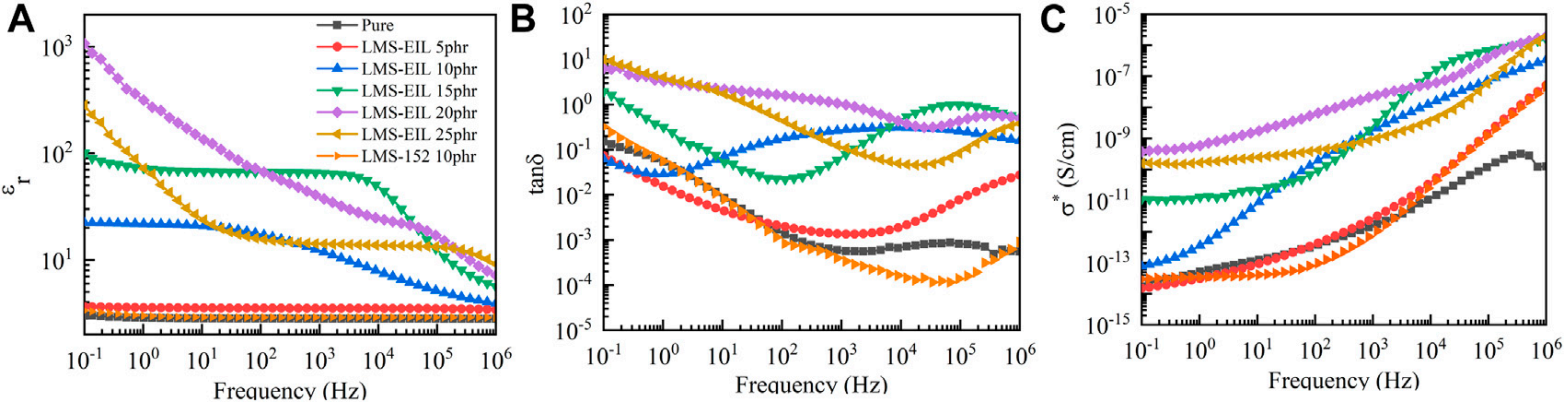
**(A)** Relative dielectric permittivity (ε_r_), **(B)** loss tangent (tanδ), and **(C)** complex conductivity (σ^*^) of elastomers with different concentrations of LMS-EIL at room temperature.

The effect of LMS-EIL concentration on the dielectric properties of composite elastomers was investigated using dielectric spectroscopy. As the comparison with the pure elastomer in Figure 3A clearly illustrates, the elastomer modified with LMS-EIL displays a higher ε_r_ across the whole test frequency range. The ε_r_ at 10^−1^ Hz of the elastomers increased initially from 3.7 to ∼1,000 with increasing LMS-EIL content from 5 to 20 phr, then decreased significantly to 280 for the 25 phr sample, as detailed in Supplementary Table S2. The increasing relative dielectric permittivity of the LMS-EIL-modified elastomers is due to the increased concentration of high-permittivity IL, while the subsequent decrease results from the aggregation of LMS-EIL in the elastomer matrix, as shown in Figure 1. Moreover, the elastomers with 10 and 15 phr LMS-EIL both display noticeable ε_r_ plateaus at frequencies below 10^2^ Hz and 10^4^ Hz, respectively—consistent with the dielectric behavior of the LMS-EIL additive shown in Figure 2D. As noted in Figure 3A and Supplementary Table S2, the ε_r_ of the elastomer with 10 phr of LMS-EIL increased 6.7-fold at 10^−1^ Hz compared to that of the elastomer with LMS-152—indicating that, as expected, it is more efficient to modify elastomers with high-permittivity LMS-EIL than with the pristine chloropropyl silicone oil LMS-152.

At 10^−1^ Hz, the tanδ values of elastomers with 5 and 10 phr LMS-EIL were 0.07 and 0.06, respectively—lower than that of both the pure elastomer and the elastomer with LMS-152. At 10^6^ Hz, however, elastomers with LMS-EIL showed higher tanδ values compared to both the pure elastomer and the elastomer with LMS-152. This can be explained by the fact that, as seen in Figure 2E, LMS-EIL displays a low loss tangent at low frequency, thus decreasing the tanδ of the resulting elastomers. Nevertheless, when the LMS-EIL content was further increased to more than 10 phr, the tanδ of the resulting elastomers increased above that of both the pure elastomer and the elastomer with LMS-152 at 10^−1^ Hz.

Figure 3C shows the complex conductivity of the studied elastomers. It is notable that σ^*^ of the elastomers modified with LMS-EIL increased with increasing LMS-EIL content for the range of 0–15 phr; above 15 phr, however, the elastomers showed inconsistent σ^*^ values due to the lack of homogeneity of the elastomers (Figure 1). Due to the higher σ^*^ of LMS-EIL compared to LMS-152, the σ^*^ of the elastomer with 10 phr LMS-EIL was 2.6 and 7.6 times higher than that of the corresponding elastomer with LMS-152 at 10^−1^ and 10^6^ Hz, respectively, as shown in Figure 2F. Importantly, while the elastomer with 10 phr LMS-EIL had the highest σ^*^ at 10^−1^ Hz, both the pure elastomer and the elastomer with LMS-152 displayed σ^*^ values on the same order of magnitude, indicating that the elastomer with 10 phr LMS-EIL possesses extremely low complex conductivity and thus significant potential as a dielectric material.

### Mechanical Properties of PDMS Elastomers

**FIGURE 4:**
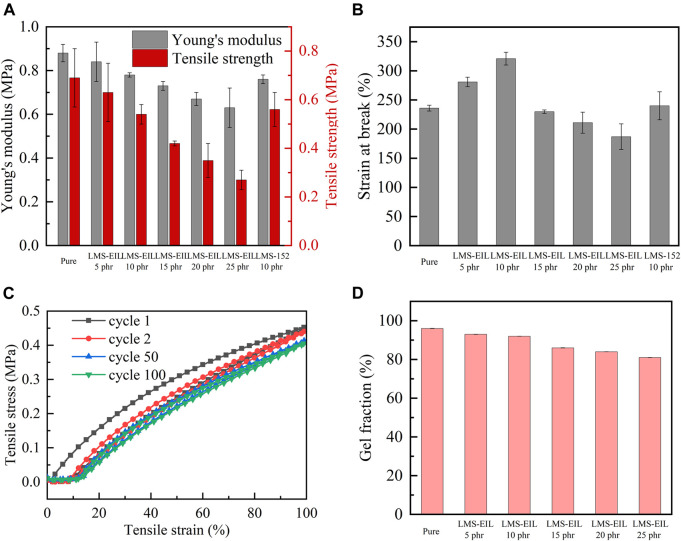
**(A)** Young’s modulus and tensile strength of PDMS elastomers; **(B)** strain at break of PDMS elastomers; **(C)** cycle stress-strain curves of PDMS elastomer with 10 phr LMS-EIL; **(D)** gel fractions of pure elastomer and PDMS elastomers with different LMS-EIL concentrations.

In flexible capacitive pressure sensors, the relative dielectric permittivity and softness of the dielectric layer both decisively influence capacitance and sensitivity. Tensile testing was therefore performed to evaluate the Young’ modulus as well as tensile strength and strain of PDMS elastomers with different LMS-EIL contents. As shown in Figure 4A and Supplementary Table S3, both Young’ modulus and tensile strength were highest in the pure elastomer, as expected, since both of these values tend to decrease with increasing LMS-EIL content due to softening effect of the additive on the elastomer/dilution of the network (Shivapooja et al., 2016); this effect is evident from the decrease in shear modulus with increasing LMS-EIL content shown in Supplementary Figure S5. A decrease in both Young’s and shear moduli—i.e., softening—makes it easier to alter the shape of dielectric layer in order to improve sensor sensitivity. The elastomer with 10 phr LMS-EIL possessed only a slightly higher Young’s modulus (0.78 MPa) and slightly lower tensile strength (0.54 MPa) compared to the elastomer with the same concentration of LMS-152 (0.76 and 0.56 MPa, respectively), indicating no significant difference in softening effect between the two additives with and without IL. Figure 4B shows the strain at break for all elastomers studied. The elastomer with 10 phr LMS-EIL exhibited a strain at break of ∼321%, higher than that of the pure elastomer as well as the elastomers with 5 or 15–25 phr LMS-EIL. These results indicate that gains in elastomer strain at break resulting from the addition of LMS-EIL are mitigated at concentrations above 10 phr due to the stress concentrations at aggregation areas in the elastomer matrix. Compared to the other composites with additives (Table 1), the excellent combination of dielectric and mechanical properties achieved by the elastomer with 10 phr LMS-EIL can be attributed to the high permittivity, softness and high compatibility of the IL-grafted functional oil.

**TABLE 1:** Comparison of dielectric and mechanical properties of composite elastomer with 10 phr LMS-EIL against other elastomeric composites reported in literature.

**Sample**	**Filler**	**Filler state**	**Filler loading**	**Relative permittivity@10^−1^ Hz**	**Young’s modulus (MPa)**	**Strain at break (%)**	**References**
PDMS	LMS-EIL	Liquid	10 phr	22	0.78	321	Current work
Liquid silicone rubber	TiO_2_	Solid	35% by weight	4.9	0.85	190	Yu and Skov (2015)
PDMS	BmimSbF_6_	Liquid	70 phr	10	0.2	60	Liu et al. (2020b)
PDMS	[PrMIm]	Liquid	40% by weight	16	0.5	355	Shi et al. (2021)
	[NTf_2_] and FEC mixture						
PDMS (Sylgard 184)	Glycerol	Liquid	50% by volume	16	0.33	116	Liu et al. (2020a)

As shown in Figure 4C, the cyclic loading-unloading curves indicate that the elastomer with 10 phr LMS-EIL has a low mechanical hysteresis; further, the fact that the mechanical hysteresis loop becomes more stable after the first cycle suggests excellent long-term mechanical stability. Indeed, the elastomer with 10 phr LMS-EIL maintained 94% elastic energy after 100 loading-unloading cycles, as shown in Supplementary Figure S6 and Supplementary Table S4. Overall, this elastomer displays excellent mechanical properties due to a combination of inherent softness, sufficiently high strain at break and elasticity, and long-term stability.

### Gel Fraction of PDMS Elastomers

Gel fractions of LMS-EIL-modified elastomers were investigated via swelling experiments in order to determine the amount of bonded (gel fraction) and non-bonded species in the elastomer networks. Figure 4D and Supplementary Table S5 list the gel fractions of the elastomers studied: gel fraction decreased with increasing LMS-EIL content, indicating that a significant amount of unreacted PDMS chain remained in the PDMS matrix in elastomers with high concentrations of LMS-EIL. This may be due to a slight inhibitory effect of IL on the Sn catalyst (Skov and Yu, 2018; Liu et al., 2019), attenuating its catalytic efficiency in the condensation curing reaction. The elastomer with 10 phr LMS-EIL had a gel fraction of 92%, within acceptable limits for silicone networks and comparable to our previous results for soft elastomers (Madsen et al., 2018; Liu et al., 2019). This elastomer also displayed a relative viscous loss of ∼2% at 10^2^ Hz (Supplementary Figure S5), indicating that it maintained a high degree of crosslinking and mechanical integrity. Therefore, the silicone composite presented a little viscoelastic effect in cyclic loading and unloading (Figure 4C).

### Sensor Performance

**FIGURE 5:**
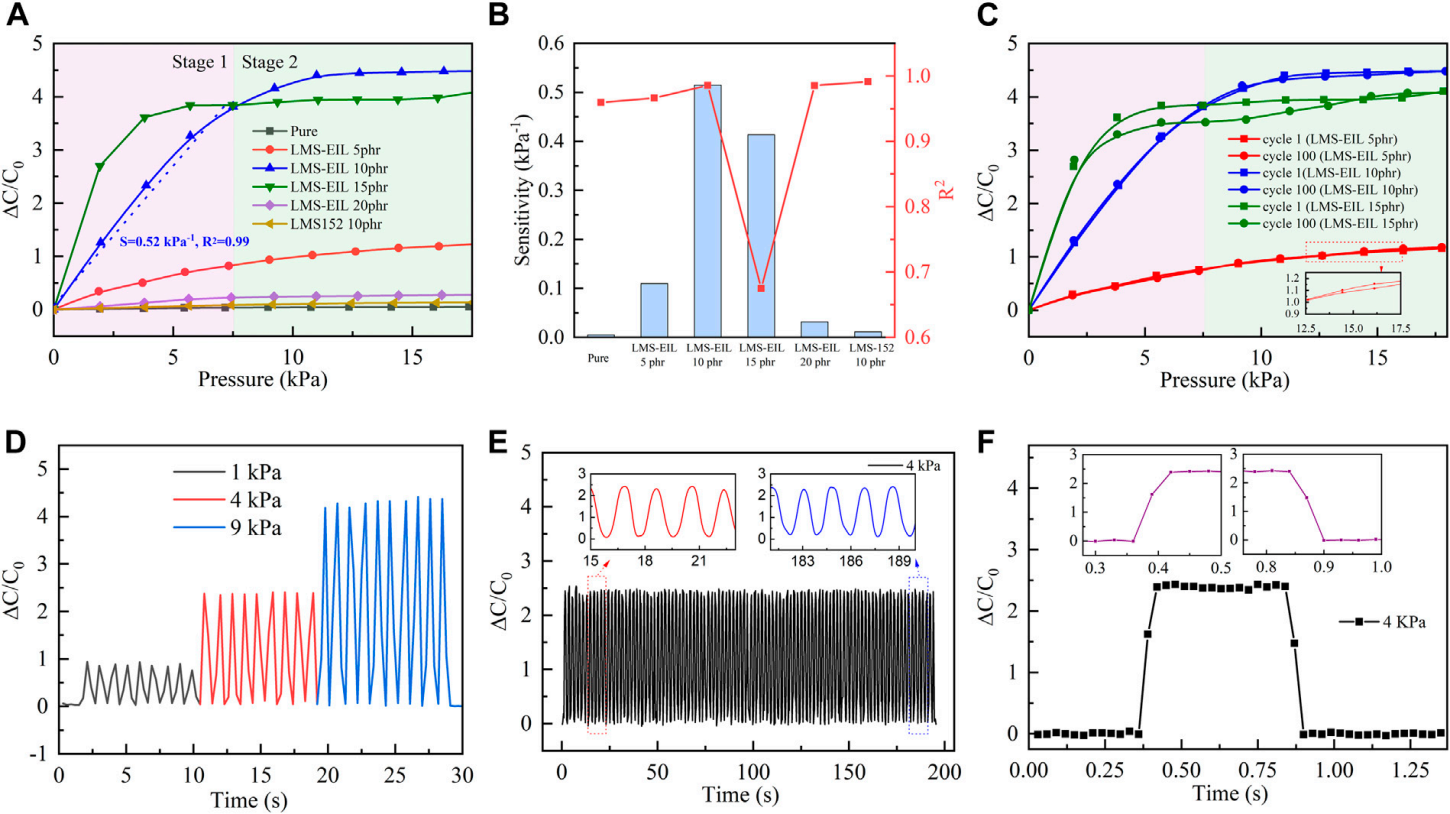
**(A)** Relative capacitance change–pressure curves for capacitive pressure sensors based on PDMS elastomers; **(B)** sensitivity and R^2^ of sensors in the pressure range of 0–7.5 kPa; **(C)** relative capacitive change for sensors with 5, 10, and 15 phr LMS-EIL at cycle 1 and cycle 100; **(D)** dynamic response of sensor with 10 phr LMS-EIL under 1, 4, and 9 kPa; **(E)** durability of 10 phr LMS-EIL sensor under 4 kPa of pressure; **(F)** response and recovery times of 10 phr LMS-EIL sensor under 4 kPa of pressure.

Pressure sensors were prepared using LMS-EIL-modified PDMS elastomers as dielectric layers and their sensing performance was evaluated. The procedure for preparation and characterization is shown in Supplementary Figure S8. The photographs for elastomer with 10 phr LMS-EIL and the obtained capacitor are shown in Supplementary Figure S9. Figure 5A shows the relative capacitive change (ΔC/C_0_) as a function of applied pressure (P) for sensors utilizing pure elastomer as well as elastomers with various LMS-EIL and LMS-152 additions: the higher ΔC/C_0_ observed for the sensor with 10 phr LMS-EIL compared to the sensor with LMS-152 can be attributed to the LMS-EIL elastomer’s higher relative dielectric permittivity (Figure 3A). The ΔC/C_0_ of sensors utilizing LMS-EIL initially increases as a result of the increased ε_r_ and decreased Y of the elastomers before decreasing once a concentration threshold is reached due to LMS-EIL aggregation (Figures 1D,E).

The sensitivity of a capacitive pressure sensor (S) is defined by the equation: S = ((∆C/C_0_))/P, where ΔC is the change of capacitance and C_0_ is the capacitance without applied pressure. According to this equation, sensitivity increases with greater variation in the relative capacitive change per unit pressure. Figure 5A shows the sensors’ relative capacitive change at low and high pressure (Stage 1 and Stage 2). At Stage 1, ΔC/C_0_ changes rapidly with increasing pressure in the range of 0–7.5 kPa (highlighted in purple). Pressure sensor sensitivities were calculated by fitting ΔC/C_0_ linearly from 0 to 7.5 kPa, and the resulting sensitivities and R-squared values are summarized in Figure 5B and Supplementary Table S6. Sensors with LMS-EIL displayed increasing sensitivity before beginning to decrease at LMS-EIL concentrations as a result of the more inhomogeneous composites. The combination of low Y, high ɛ_r_ and morphology homogeneity in the elastomer with 10 phr LMS-EIL produced the sensor with the highest sensitivity of 0.51 kPa^−1^, which is 100 and 51 times higher, respectively, compared to the sensors based on pure elastomer (0.005 kPa^−1^) and a 10 phr LMS-152 addition (0.01 kPa^−1^). The promising sensitivity and linear sensing pressure range of sensor with 10 phr LMS-EIL compare well against other recent reports of sensors with additives (Table 2). In addition, the sensor with 10 phr LMS-EIL shows 51-fold higher sensitivity as compared to that of the sensor with 10 phr LMS-152, while the relative permittivity just presents a 6.7 times increase (Figure 3A). The significant increase in pressure sensitivity may be due to the higher relative permittivity change of the potentially nanoporous structure with compression. The R-squared value from the linear regression for the sensor with 10 phr LMS-EIL is 0.99, indicating that the sensor displays almost perfectly linear response in the pressure range of 0–7.5 kPa due to the excellent dispersion of LMS-EIL additive in the dielectric layer (Figure 1C) and the robust mechanical properties (Figure 4C). The importance of dispersion quality and robust mechanical properties is further stressed by the sensor with 15 phr LMS-EIL which exhibits a really low R-squared value (*R* ^2^ = 0.67) compared to the 10 phr sample. At Stage 2 (highlighted in olive green), sensors were subjected to higher pressure (7.5–17.5 kPa). The sensors with both LMS-EIL and LMS-152 exhibited much lower sensitivity under higher pressure—typical behavior for flexible capacitive sensors that experience an increase in the elastic resistance (Yin et al., 2018; Li et al., 2020).

**TABLE 2:** Comparison of sensing performance of sensor with 10 phr LMS-EIL against other elastomeric composites.

**Dielectric layer**	**Sensitivity (kPa^−1^)**	**Linear sensing range (kPa)**	**Reference**
PDMS composites with 10 phr LMS-EIL	0.051	0–8	Current work
PDMS composites with ionic liquid and keratin as filler	0.037	0–8	Liu et al. (2020b)
PDMS foam with glycerol as filler	0.05	0–6	Liu et al. (2020a)
EMIMTFSI−PDMS self-enclosed composites	0.03	0–5	Ankit et al. (2020)
MWCNT and TPU loaded silicone rubber	0.032	0–10	Lee et al. (2019)

The relative capacitance change of elastomers with LMS-EIL became saturated with increasing pressure beyond a certain pressure, such as 10 kPa for elastomer with 10 phr LMS-EIL. This may be due to the elastomer with LMS-EIL behave like a nano-porous foam that has the included oil being squeezed out above a certain pressure threshold. However, the SEM images in Figure 1 do not show any foam-like structure, most likely because the LMS-EIL and PDMS chains are fully miscible, and thus they cannot be observed on the micro-scale. In addition, the saturation pressure (10 kPa) of elastomer with 10 phr LMS-EIL is relatively small as compared to the Young’s modulus (0.78 MPa, Figure 4A) of the silicone elastomeric composite. The Young’s modulus was determined by tension testing, while the applied pressure when sensor testing is related to the compressive modulus.

The cyclic relative capacitive change for the pressure sensors with 5, 10 and 15 phr LMS-EIL at various pressures ranging from 0 to 18 kPa is shown in Figure 5C. The amplitude of this change is nearly unaltered after 100 loading-unloading cycles at two stages for the sensors with 5 and 10 phr LMS-EIL, while the sensor with 15 phr LMS-EIL now exhibits serious deviation. This non-coincident cyclic response behavior is the result of LMS-EIL aggregation in the elastomer (Figure 1D).

The response of the sensor constructed with 10 phr LMS-EIL under 1, 4, and 9 kPa is shown in Figure 5D. This sensor shows a stable capacitive response and excellent repeatability under nine loading-unloading cycles at each pressure. It also exhibits a rapid corresponding capacitive response to a step pressure, indicating outstanding pressure recognition performance.

The durability of this same sensor was evaluated via a cyclic test of 100 cycles at a pressure of 4 kPa. As shown in Figure 5E, due to the excellent elasticity of the PDMS elastomer (Figure 4C), no obvious fatigue or degradation occurred during the 100 loading-unloading cycles. This continuously stable and rapid response under cyclic testing indicates that the sensor with 10 phr LMS-EIL has sufficient durability for real-time applications. However, due to the limited testing instruments, we just provide general data to support the long-term stability of the sensor. Some tests, such as thermal effects including diffusion and radiation, are also important factors to the long-term stability of the sensor, and they deserve further study in the future.

Figure 5F shows the relative capacitive change curve of the sensor with 10 phr LMS-EIL at a pressure of 4 kPa in a single loading-unloading cycle. The ΔC/C_0_ presents negligible fluctuation during both loading and unloading. Furthermore, ΔC/C_0_ increases immediately from 0 to 2.0 when pressure is applied to the sensor. Conversely, ΔC/C_0_ returns quickly to baseline upon removal of the applied pressure. As shown in the magnified response and relaxation curve in Figure 5F, both response and recovery times are around 60 ms—faster than those of sensors with complicated, prefabricated structure to increase their sensitivity (around 100 ms) (Ma et al., 2018). This combination of stability with fast response and recovery means that the 10 phr LMS-EIL sensor possesses high potential as healthcare and wearable applications.

## Conclusion

A novel approach for generating high-permittivity elastomers has been developed via the incorporation of functional silicone oil, which is chemically grafted a priori using ionic liquid. The functional silicone oil LMS-EIL provides significantly increased relative dielectric permittivity (almost 10^5^ times) and decreased loss tangent (∼30 times) compared to chloroprpyl silicone at 10^−1^ Hz. A composite elastomer incorporated with 10 phr of LMS-EIL exhibits homogeneous morphology, low Young’s modulus (0.78 MPa), high stretchability (∼321%), and significantly improved relative dielectric permittivity (ε_r_ ∼22 at 10^−1^ Hz) compared to the pure elastomer film. This composite elastomer is also well cross-linked, stable during long-term use, and easy to fabricate. What is more, a flexible pressure sensor constructed using this composite elastomer as the dielectric layer demonstrated fast response (∼60 ms), good durability (no fatigue observed after 100 cyclic deformations), and a 50-fold increase in sensitivity over pure silicone (0.51 kPa^−1^). Preparation of high-permittivity elastomers by incorporating IL-grafted silicone oil has excellent potential for industrial applications involving high performance flexible pressure sensors.

## Data Availability

The original contributions presented in the study are included in the article/Supplementary Files, further inquiries can be directed to the corresponding authors.
