# Supplementary material for: Highly Sensitive Flexible Pressure Sensors Enabled by Mixing of Silicone Elastomer With Ionic Liquid-Grafted Silicone Oil
Source: Front Robot AI. 2021 Sep 20;8:737500. doi: 10.3389/frobt.2021.737500 (PMC8488264; doi:10.3389/frobt.2021.737500)
Supplement: Supplementary file 1 [file Table1.docx]

**Supplementary Information**

**Highly sensitive flexible pressure sensors enabled by mixing of silicone elastomer with ionic liquid-grafted silicone oil**

Zhaoqing Kang^a,b^, Yi Nie^b^, Liyun Yu^a^, Suojiang Zhang*^b^, Anne Ladegaard Skov*^a^

^a^Danish Polymer Center, Department of Chemical and Biochemical Engineering, Technical University of Denmark, Kgs. Lyngby, Denmark;

^b^CAS Key Laboratory of Green Process and Engineering, Beijing Key Laboratory of Ionic Liquids Clean Process, State Key Laboratory of Multiphase Complex Systems, Institute of Process Engineering, Chinese Academy of Sciences, Beijing, China.

Corresponding Authors:

Suojiang Zhang ─ E-mail: sjzhang@ipe.ac.cn; ORCID: https://orcid.org/0000-0002-9397-954X

Anne Ladegaard Skov ─ E-mail: al@kt.dtu.dk; ORCID: http://orcid.org/0000-0003-1223-6638

**Figure S1:** FT-IR spectra for pure PDMS elastomer, PDMS elastomer with 20 phr LMS-EIL before extraction, and PDMS elastomer with 20 phr LMS-EIL after extraction.

**Table S1:** Dielectric properties of LMS-EIL and LMS-152.

| Sample | ε_r_ @ 10^-1^ Hz | ε_r_ @ 10^6^ Hz | tanδ @ 10^-1^ Hz | tanδ @ 10^6^ Hz | σ^*^ @ 10^-1^ Hz | σ^*^ @ 10^6^ Hz |
| --- | --- | --- | --- | --- | --- | --- |
| LMS-152 | 10.6 | 3.88 | 59.2 | 5.5×10^-5^ | 3.5×10^-11^ | 1.5×10^-6^ |
| LMS-EIL | 9.6×10^5^ | 5.2 | 2.0 | 9.2 | 1.2×10^-7^ | 2.7×10^-5^ |


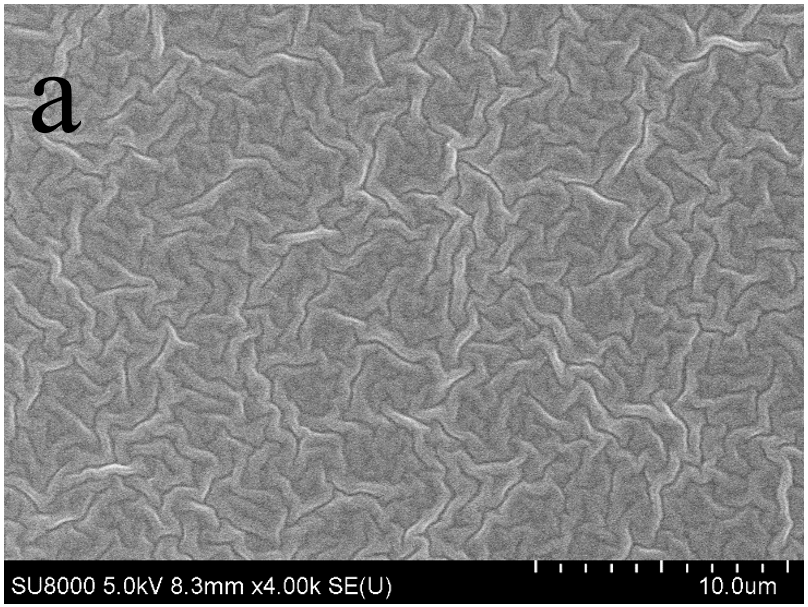

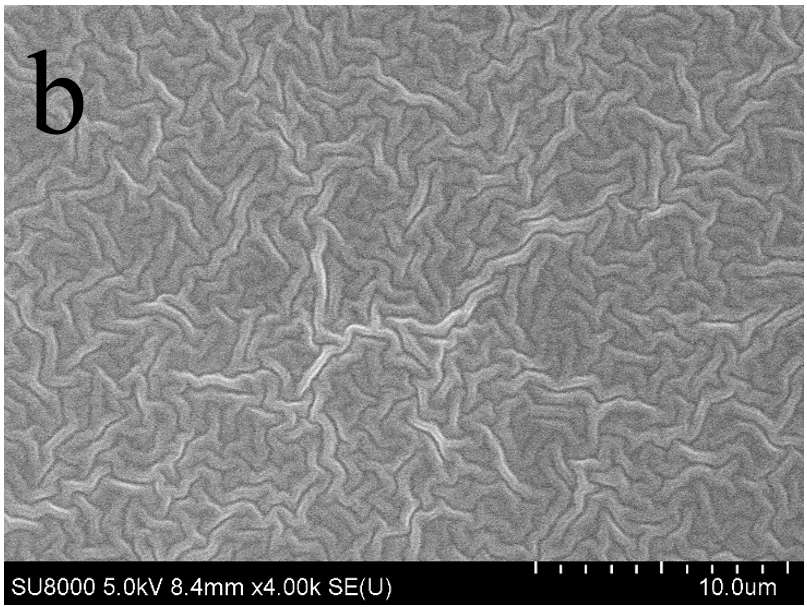

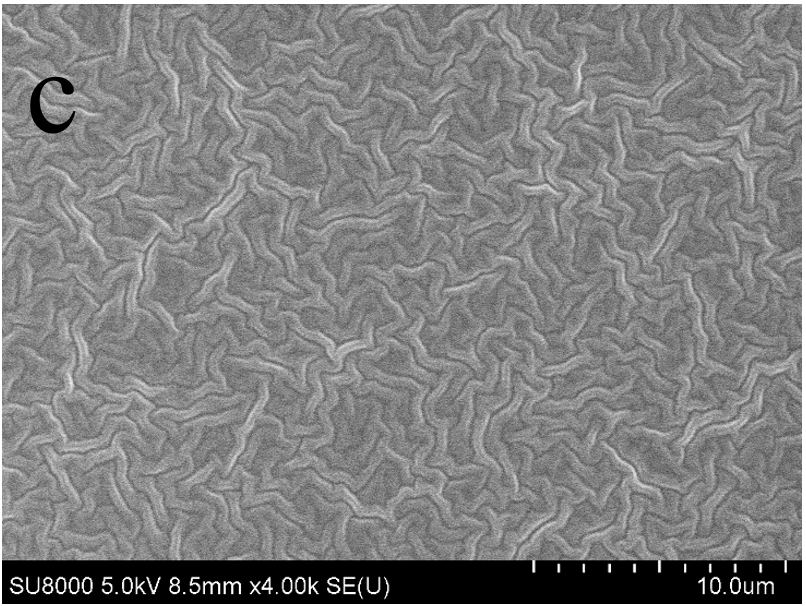

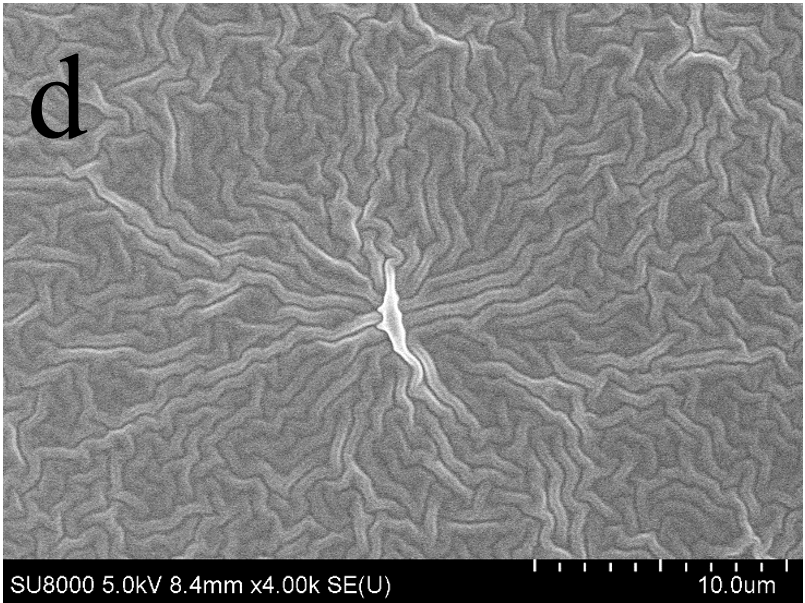

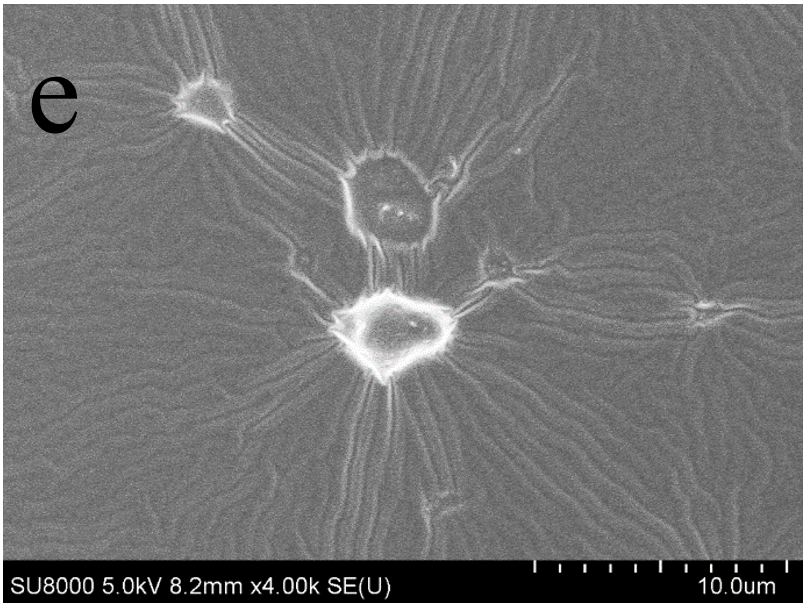

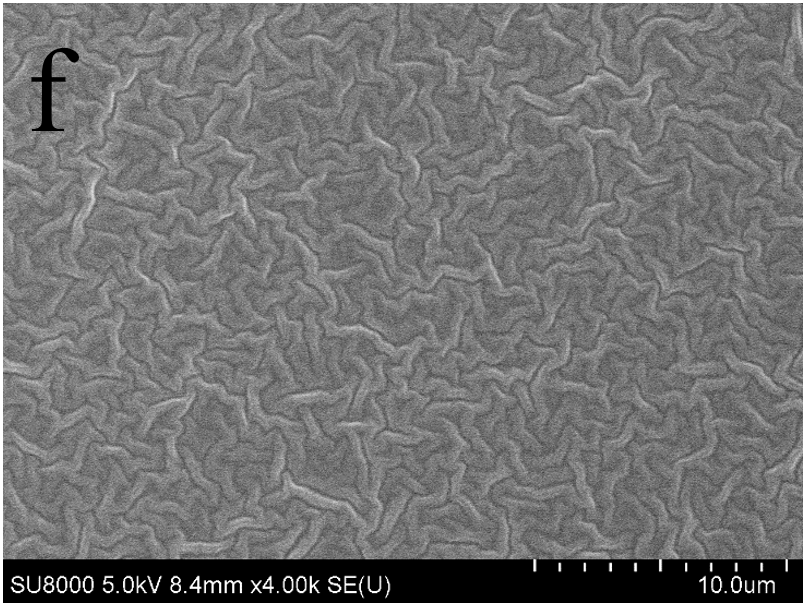


**Figure S2**: Magnified FE-SEM images of PDMS elastomers: (a) pure elastomer, (b) elastomer with 5 phr LMS-EIL, (c) elastomer with 10 phr LMS-EIL, (d) elastomer with 15 phr LMS-EIL, (e) elastomer with 20 phr LMS-EIL, and (f) elastomer with 10 phr LMS-152.


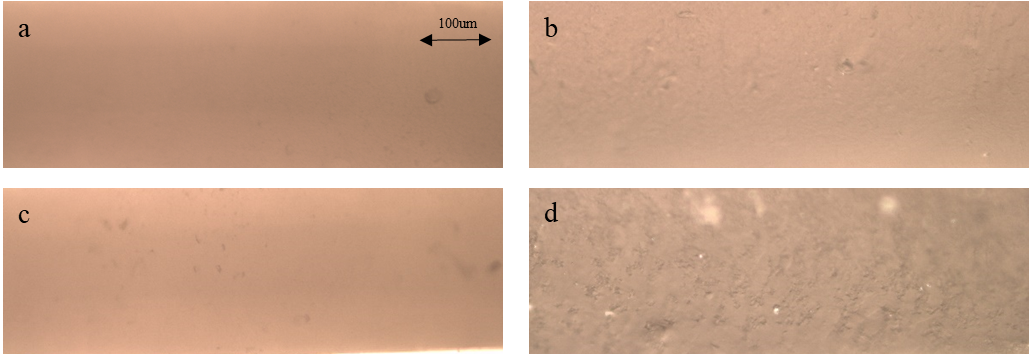


**Figure S3**: Optical cross sectional images of elastomers: (a) pure elastomer, (b) elastomer with 5 phr LMS-EIL, (c) elastomer with 10 phr LMS-EIL, and (d) elastomer with 15 phr LMS-EIL.

**
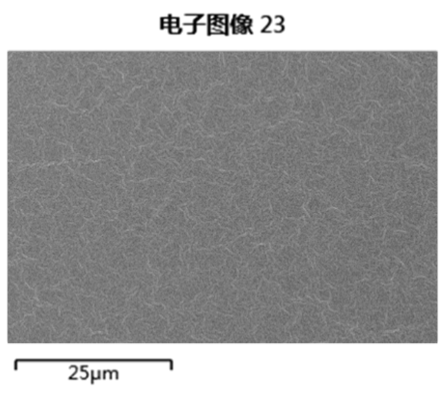

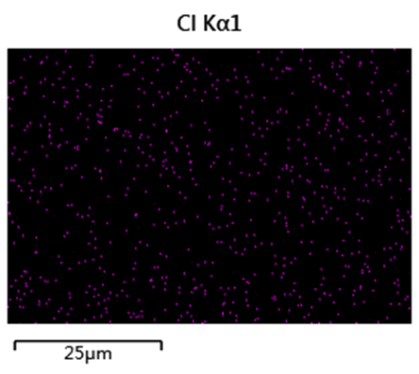
**
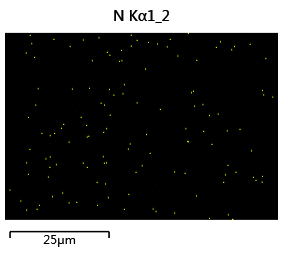


**Figure S4:** EDS images of PDMS elastomers with 10 phr LMS-EIL.

**Table S2:** Dielectric properties of elastomer with LMS-EIL and LMS-152.

| Sample | ε_r_ @ 10^-1^ Hz | ε_r_ @ 10^6^ Hz | tanδ @ 10^-1^ Hz | tanδ @ 10^6^ Hz | σ^*^ @ 10^-1^ Hz | σ^*^ @ 10^6^ Hz |
| --- | --- | --- | --- | --- | --- | --- |
| Pure | 2.98 | 2.79 | 0.15 | 5.6×10^-4^ | 1.75×10^-14^ | 1.25×10^-10^ |
| LMS-EIL 5phr | 3.65 | 3.41 | 0.07 | 0.02 | 1.49×10^-14^ | 5.18×10^-8^ |
| LMS-EIL 10phr | 22.3 | 3.90 | 0.06 | 0.16 | 7.70×10^-14^ | 3.41×10^-7^ |
| LMS-EIL 15phr | 100 | 5.61 | 1.96 | 0.50 | 1.09×10^-11^ | 1.57×10^-6^ |
| LMS-EIL 20phr | 1068 | 7.16 | 6.29 | 0.49 | 3.74×10^-10^ | 1.95×10^-6^ |
| LMS-EIL 25phr | 282 | 9.16 | 10.0 | 0.38 | 1.58×10^-10^ | 1.93×10^-6^ |
| LMS-152 10phr | 3.34 | 2.89 | 0.59 | 6.7×10^-4^ | 2.98×10^-14^ | 4.51×10^-8^ |

**Table S3:** Mechanical properties of the elastomers with different amounts of LMS-EIL.

| Sample | Y (MPa) | Tensile strain (%) | Tensile stress (MPa) |
| --- | --- | --- | --- |
| Pure | 0.88±0.04 | 236±5 | 0.69±0.12 |
| LMS-152-10 phr | 0.76±0.02 | 240±24 | 0.56±0.07 |
| LMS-EIL-5phr | 0.84±0.09 | 281±8 | 0.63±0.12 |
| LMS-EIL-10phr | 0.78±0.01 | 321±11 | 0.54±0.04 |
| LMS-EIL-15phr | 0.73±0.02 | 230±3 | 0.42±0.01 |
| LMS-EIL-20phr | 0.67±0.03 | 211±18 | 0.35±0.07 |
| LMS-EIL-25phr | 0.63±0.09 | 187±22 | 0.27±0.04 |

**Figure S5:** Shear modulus (G′) and relative viscous loss (tanδ) of the PDMS elastomers measured at 1% strain and room temperature.

**Figure S6:** Cyclic load-displacement curves of PDMS elastomers: (a) pure elastomer, (b) elastomer with 10 phr LMS-EIL, and (c) elastomer with 20 phr LMS-EIL.

Figure S7 shows the loading-displacement curves of an elastic material, the total energy consisted of elastic and inelastic energy is stored upon deformation. The elastic energy is calculated by the area under the unloading curve, and is recoverable. As the area under the unloading curve is less than that of the loading curve, which means some of the energy put into the material was transferred as waste energy (inelastic energy), like heat. The elastic energy function (Φ) is used to evaluate the hysteresis of materials as noted in Table S4. Φ is obtained by dividing the area under the unloading curve (elastic strain energy) with the area under the loading curve (total energy). For an ideal elastomer, Φ equals 1. ^1,2^

**
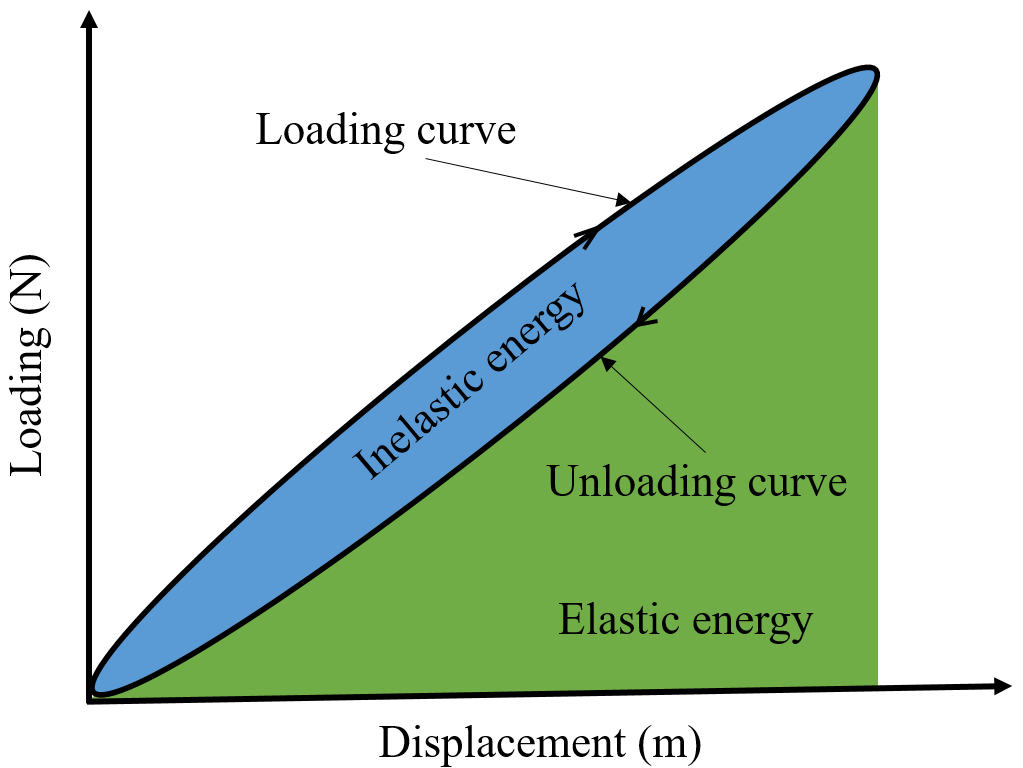
**

**Figure S7:** Determination of hysteresis of materials.

**Table S4:** Stress (σ) under the strain of 10% and elastic energy function (Φ) of PDMS elastomers in different deformation cycles: (a) pure elastomer, (b) elastomer with 10 phr LMS-EIL, and (c) elastomer with 20 phr LMS-EIL.

| Sample | Cycle | σ (MPa) | Φ (elastic/total energy) |
| --- | --- | --- | --- |
| Pure | Cycle 1 | 0.52 | 83.4 |
|  | Cycle 2 | 0.51 | 97.0 |
|  | Cycle 50 | 0.48 | 98.5 |
|  | Cycle 100 | 0.48 | 98.4 |
| LMS-EIL 10phr | Cycle 1 | 0.45 | 82.2 |
|  | Cycle 2 | 0.44 | 92.1 |
|  | Cycle 50 | 0.42 | 94.0 |
|  | Cycle 100 | 0.41 | 94.0 |
| LMS-EIL 20phr | Cycle 1 | 0.35 | 71.6 |
|  | Cycle 2 | 0.34 | 86.7 |
|  | Cycle 50 | 0.32 | 90.0 |
|  | Cycle 100 | 0.33 | 90.9 |

**Table S5:** Gel fractions of the PDMS elastomers.

| Sample | Gel fraction (%) |
| --- | --- |
| Pure | 96±0.06 |
| LMS-EIL-5phr | 93±0.03 |
| LMS-EIL-10phr | 92±0.01 |
| LMS-EIL-15phr | 87±0.04 |
| LMS-EIL-20phr | 83±0.01 |
| LMS-EIL-25phr | 80±0.04 |


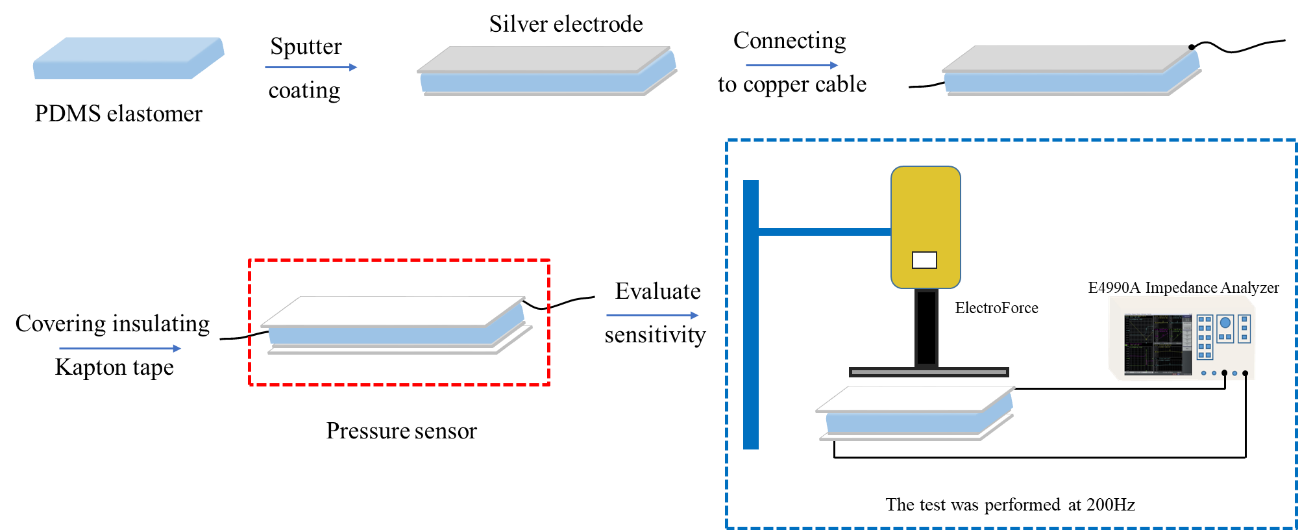


**Figure S8:** Preparation and characterization procedures of the pressure sensors.


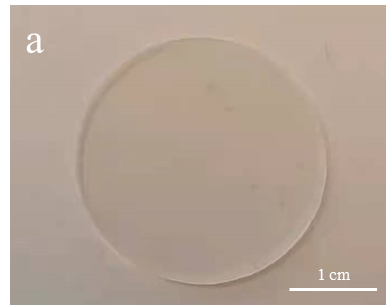

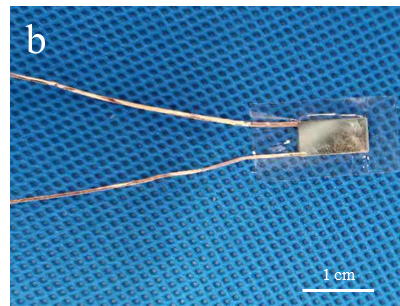


**Figure S9:** Photographs of elastomer with 10 phr LMS-EIL (a) and the obtained sensor with dimensions of 10 mm length × 5 mm width × 1 mm thickness (b).

**Table S6:** Sensitivity and R^2^ of the PDMS elastomers.

| Sample | Sensitivity (kPa^-1^) (0-7.5 kPa) | Fitting equation | R^2^ |
| --- | --- | --- | --- |
| Pure | 0.0051 | y = 0.0051x - 0.0016 | 0.9594 |
| LMS-EIL 5 phr | 0.1098 | y = 0.1098x + 0.0699 | 0.9663 |
| LMS-EIL 10 phr | 0.5147 | y = 0.5147x + 0.1744 | 0.9856 |
| LMS-EIL 15 phr | 0.4133 | y = 0.4133x + 1.0670 | 0.6748 |
| LMS-EIL 20 phr | 0.0316 | y = 0.0316x + 0.0018 | 0.9854 |
| LMS-152 10phr | 0.0112 | y = 0.0112x + 0.0022 | 0.9913 |

**References**

S1. J. Vaicekauskaite, P. Mazurek, S. Vudayagiri and A. L. Skov, J. Mater. Chem. C, 2020, **8**, 1273-1279.

S2. C. T. Lachowicz, Int. J. Fatigue, 2001, **23**, 643-652.
