# Supplementary figures and images for: Highly Sensitive Flexible Pressure Sensors Enabled by Mixing of Silicone Elastomer With Ionic Liquid-Grafted Silicone Oil
Source: Front Robot AI. 2021 Sep 20;8:737500. doi: 10.3389/frobt.2021.737500 (PMC8488264; doi:10.3389/frobt.2021.737500)

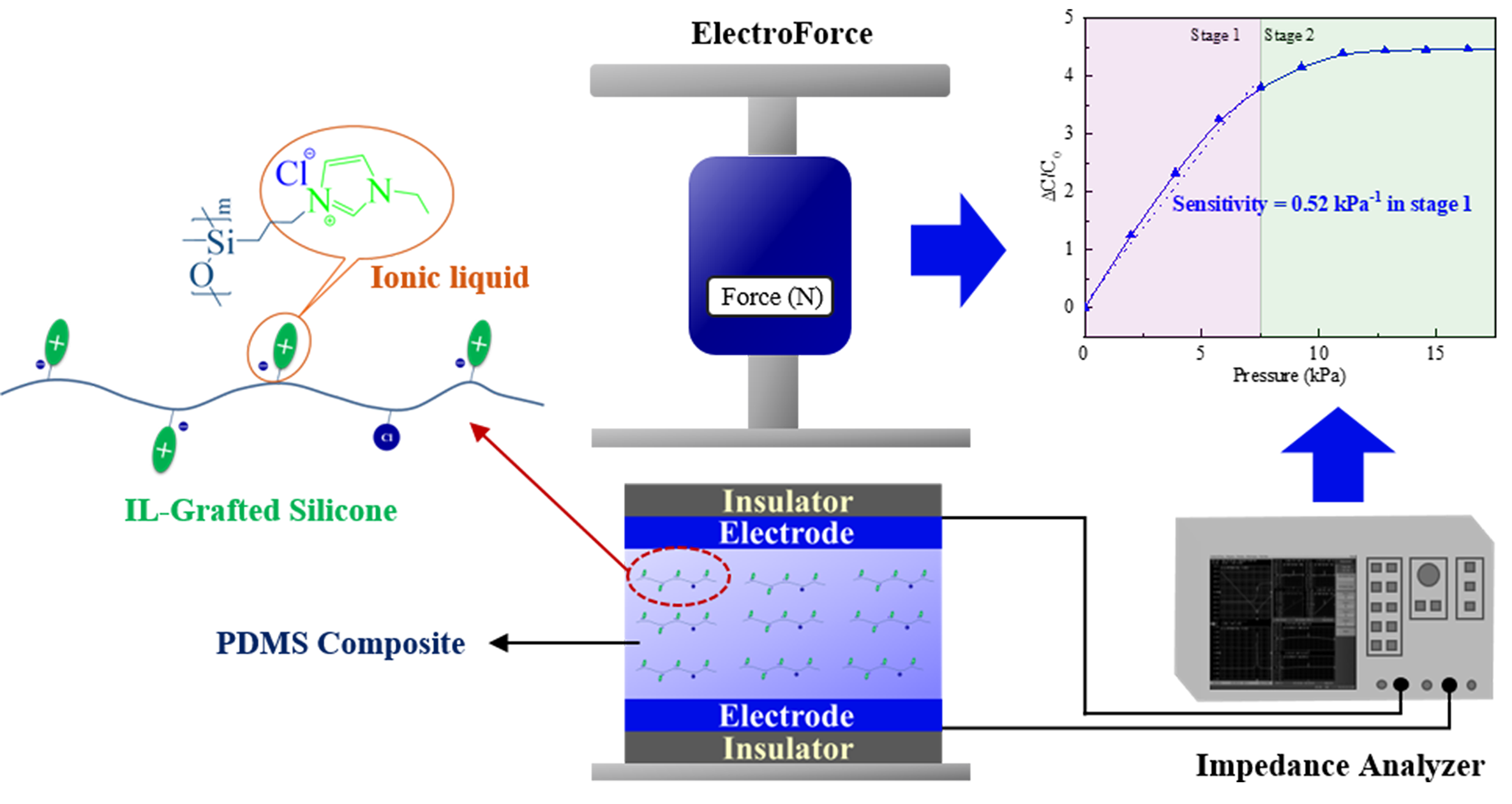

Supplement: Supplementary file 2 [file Image1.TIF]
